# Nuclear Intron Sequence Variation of the *Bulinus globosus* Complex (Mollusca: Planorbidae): Implications for Molecular Systematic Analyses

**DOI:** 10.3390/biology14010053

**Published:** 2025-01-10

**Authors:** Chairat Tantrawatpan, Kotchaphon Vaisusuk, Chrysantus M. Tanga, Warayutt Pilap, Naruemon Bunchom, Ross H. Andrews, Tongjit Thanchomnang, Wanchai Maleewong, Weerachai Saijuntha

**Affiliations:** 1 Division of Cell Biology, Department of Preclinical Sciences, Faculty of Medicine, and Center of Excellence in Stem Cell Research and Innovation, Thammasat University, Rangsit Campus, Pathum Thani 12120, Thailand; talent3003@yahoo.com; 2 Department of Veterinary Technology, Faculty of Agricultural Technology, Rajabhat Maha Sarakham University, Maha Sarakham 44000, Thailand; kotchaphontik@gmail.com; 3 Plant Health Theme, International Centre of Insect Physiology and Ecology, Nairobi 00100, Kenya; ctanga@icipe.org; 4 Walai Rukhavej Botanical Research Institute, Mahasarakham University, Maha Sarakham 44150, Thailand; warayutt@msu.ac.th; 5 Department of Tropical Medicine and Malaria, Research Institute, National Center for Global Health and Medicine, 1-21-1 Toyama, Shinjuku-Ku, Tokyo 162-8655, Japan; aoy_narumon@hotmail.com; 6 Department of Surgery & Cancer, Faculty of Medicine, Imperial College London, South Kensington Campus, London SW7 2AZ, UK; rhandrews@gmail.com; 7 Biomedical Science Research Unit, Faculty of Medicine, Mahasarakham University, Maha Sarakham 44000, Thailand; tongjit.t@msu.ac.th; 8 Department of Parasitology, Faculty of Medicine, and Mekong Health Science Research Institute, Khon Kaen University, Khon Kaen 40002, Thailand; wanch_ma@kku.ac.th

**Keywords:** pulmonate snail, intermediate host, schistosomiasis, heterozygosity, genetic variation, DNA recombination, species complex

## Abstract

Urinary schistosomiasis is caused by the blood fluke *Schistosoma haematobium*, which is predominantly found in Africa. The freshwater snail *Bulinus globosus* is its main intermediate host. The species that make up the *B. globosus* group are genetically complex, and their taxonomic status remains controversial. Genetic variation, heterozygosity, and DNA recombination in *B. globosus* were examined using the mitochondrial cytochrome c oxidase subunit 1 (*COI*) and the intron 3 region of the arginine kinase gene (AkInt3). A total of 81 *B. globosus* snails were collected from three different localities in Kwale County, Kenya. Genomic diversity, heterozygosity, DNA recombination, and haplotype network were calculated using AkInt3 sequences. Low polymorphism in the *COI* sequence divided *B. globosus* into six haplotypes (C1–C6). However, AkInt3 sequencing studies showed high polymorphisms, classifying 81 *B. globosus* snails into 44 haplotypes (H1–H44). These haplotypes were separated into three haplogroups (I–III). AkInt3 sequence heterozygosity was also found. DNA recombination haplotypes between haplogroups were commonly found in heterozygous samples. AkInt3 sequence studies showed high levels of genetic polymorphism and heterozygosity, supporting its use as a genetic marker for elucidating the population genetics of *B. globosus*. Furthermore, our study showed that *B. globosus* populations in Kenya form a “species complex”.

## Simple Summary

This study highlights the genetic diversity of *Bulinus globosus* in Kenya, demonstrating that the AkInt3 intron is a valuable marker for detecting detailed intra-specific genetic variation, surpassing *COI* sequences. The presence of DNA recombination between AkInt3 haplogroups suggests that cross-fertilization is a common reproductive strategy, which may reduce inbreeding effects. Additionally, evidence of potential polyploidy points to further genetic complexity, warranting more studies. The findings indicate that AkInt3 primers could aid genetic studies in other *Bulinus* species, with distinct haplogroups suggesting significant genetic diversity. Future research across Africa will be essential for understanding *B. globosus* evolution and its role in disease transmission.

## 1. Introduction

There are several species of the freshwater pulmonate snails within the genus *Bulinus* that have been recognized as intermediate hosts of the medically and veterinary important blood fluke genus *Schistosoma*, a causative agent of schistosomiasis [1]. At least 250 million people are at risk of infection since 2021, with more than 75.3 million people suffering from schistosomiasis worldwide, and of these cases, over 90% occur on the African continent [2]. The *Bulinus* snails have been divided into four species groups, namely the *B. africanus* group, *B. forskalii* group, *B. reticulatus* group, and *B. truncatus*/*tropicus* complex group [1]. Despite limited morphological divergence within species groups, there is considerable molecular divergence [3]. Within the *B. africanus* group, at least 10 species are currently recognized and are distributed throughout sub-Saharan Africa [4]. One of the most common species of the *B. africanus* group is the *B. globosus* species complex. *Bulinus globosus* (Morelet, 1866) is the sole intermediate host of *Schistosoma haematobium* (Bilharz, 1852), a causative agent of human schistosomiasis. It was estimated that 800 million people are at risk of infection. At least 10 million people had *S. haematobium*-related renal failure and schistosomiasis-related bladder cancer, resulting in an estimated mortality of 280,000 people per year in Africa [5,6].

The common habitats of *B. globosus* are a wide variety of often transient and patchily distributed freshwater habitats, such as the shores of lakes, ponds, streams, and irrigation canals [1]. Previous reports have found that the genetic structure of *B. globosus* was shaped mainly by the spatial distribution of habitats, which were influenced by spatial and temporal fluctuations in the availability of water, resulting in population bottlenecks [7]. Spatial distance also limits gene flow among *B. globosus* as a consequence of isolation-by-distance between populations [7,8]. Although *B. globosus* is a hermaphrodite that enables self-fertilization [9], it normally exhibits a mixed reproductive strategy, although they adopt only one reproductive mode at any particular time. There is evidence proposing outcrossing as a way to avoid inbreeding depression [9].

The taxonomic status of *Bulinus* snails using morphological characters within the *B. africanus* group is still controversial [3]. Thus, identifying and establishing molecular techniques and potential genetic markers may provide the basis to resolve this problem. For example, *B. globosus* and *B. nasutus* (von Martens, 1879) populations in East Africa could be distinguished by variation in the mitochondrial cytochrome c oxidase subunit 1 (*COI*) sequence [10]. Moreover, the *COI* sequence together with the nuclear ribosomal internal transcribed spacer (ITS), 18S, and 28S regions serve as effective genetic markers for differentiating and elucidating the phylogenetics of the *Bulinus* snails in the *B. africanus* group [3,4]. There are several reports on the molecular systematics, genetic diversity, and population genetic structure of the *B. globosus* complex in Africa based on microsatellite DNA analyses [7,8,11]. Previous studies found that the genetic structure of the *B. globosus* complex on a macro-scale was predominantly related to geographical distribution, such as the occurrence in the south, east, and west of Africa [3]. However, other polymorphic genetic markers, for instance, nuclear intron sequences, may provide a more comprehensive understanding of the genetic diversity, genetic structure, and phylogenetics of *B. globosus* groups distributed in Africa.

Intron regions of the arginine kinase gene have been characterized and applied as molecular markers for genetic studies in several trematodes, e.g., liver flukes *Fasciola* [12] and *Opisthorchis viveririni* (Poirier, 1886) and *Clonorchis sinensis* Looss, 1907 [13]. Moreover, the AK intron regions of several freshwater snails have been identified as potential and highly polymorphic nuclear DNA markers for genetic investigations of *Semisulcospira libertina* (Gould, 1859) snails, the intermediate host of *Paragonimus westermani* Kerbert, 1878 [14], and species of *Bithynia* snails, the intermediate host of *O. viverrini* [15]. In addition, analyses of intron sequences have the advantage of elucidating heterozygosity levels and DNA recombination between different species and/or defined genetic groups [12]. Thus, our study aims to use an intron sequence of AK to determine and define the genetic variation and heterozygosity of the *B. globosus* complex collected from different geographical localities in Kenya, Africa.

## 2. Materials and Methods

### 2.1. Sample Preparation

A total of 81 *B. globosus* snails were collected from three different localities, namely Kinango Dam (KD), Mwachinga (MC), and Maelinane (ML) in Kwale County, Kenya (Table 1). All snails were subjected to cercarial shedding by sunlight [16] and searching for schistosome cercariae before molecular analysis. *Bulinus* snails were initially identified using standard morphological criteria described by Kristensen [17] and subsequently confirmed through molecular identification using *COI* genotyping of both the snails and the shed schistosome cercariae. DNA was individually extracted from their head–foot using the E.Z.N.A.^®^ Mollusc DNA kit (Omega bio-tek, Norcross, GA, USA) following the manufacturer’s protocol, and samples were kept at –20 °C until further required.

### 2.2. PCR and DNA Sequencing

The *COI* gene (Folmer region) was amplified by polymerase chain reaction (PCR) processing using primers LCO1490 (5′-GGT CAA CAA ATC ATA AAG ATA TTG G-3′) and HCO2198 (5′-TAA ACT TCA GGG TGA CCA AAA AAT CA-3′) [18]. Meanwhile, primer pairs of BulAkInt3F (5′-TGA GGC CCT GAC CTC ACT G-3′) and BulAkInt3R (5′-TTT CTG CAT GGA GAT GAC CC-3′) were designed to anneal the flanking region of arginine kinase to amplify the intron 3 region. The PCR consisted of a 25 µL final reaction volume containing 1× Ex buffer (Takara, Shiga, Japan) with 0.2 mM each of dNTP, 0.2 µM of each forward primer, 0.625 U of Ex *Taq* DNA polymerase (Takara, Shiga, Japan), and 1 µL (~10–50 ng) of the DNA sample. The PCR conditions that were used for the amplification of both regions were: 94 °C for 4 min, followed by 35 cycles of 1 min each at 94 °C, 50 °C, and 72 °C, and final extension at 72 °C for 8 min. The amplified products were analyzed using 1.0% agarose gel electrophoresis and then cut and purified using an E.Z.N.A.^®^ Gel Purification kit (Omega bio-tek, Norcross, GA, USA). Subsequently, purified PCR products were sent for nucleotide sequencing (ATGC Co., Ltd., Pathum Thani, Thailand).

### 2.3. DNA Cloning

If heterozygosity was observed in the AkInt3 sequence of a particular sample, the purified PCR product was cloned into a pGEM-T easy vector (Promega, Madison, WI, USA), following the manufacturer’s protocol. The recombinant plasmid was introduced and propagated in *Escherichia coli* JM109 (Takara, Shiga, Japan). Four to eight white colonies from screening on Luria–Bertani (LB) agar media, containing isopropyl β-D1-thiogalactopyranoside (IPTG), 5-bromo-4-chloro-3-indolyl-β-D-galactopyranoside (X-Gal), and ampicillin, were randomly picked and cultured overnight in 2 mL of LB broth containing ampicillin (100 mg/L). The plasmid DNA was extracted using the FastGene^®^ Plasmid Mini kit (Nippon Genetics Co., Ltd., Tokyo, Japan), then cycle-sequenced at the Eurofins Genomics Company, Japan, in both directions using forward and reverse primers, M13F and M13R, respectively, as sequencing primers.

### 2.4. DNA Sequence Analyses

The DNA was assembled and manually edited using BioEdit v.7.2.6 [19]. The *COI* and AkInt3 sequences of *Bulinus* determined in this study were deposited in GenBank under the accession numbers PQ756944–PQ756949 and PQ783158–PQ783201, respectively. All *COI* sequences were subjected to a BLAST search [20] in the National Center for Biotechnology Information (NCBI) GenBank (https://blast.ncbi.nlm.nih.gov/Blast.cgi accessed on 20 July 2024) for species confirmation. Multiple alignments were performed using ClustalW [21]. The number of segregation sites (S), number of haplotypes (H), haplotype diversity (Hd), and nucleotide diversity (π) were calculated using DnaSp v.5.10.01 [22].

A phylogenetic tree was reconstructed by using *COI* sequences based on the neighbor-joining (NJ) method using the Morgan 2-parameter model [23] with a bootstrap support of 1000 replications; and using the maximum likelihood (ML) method using the general time reversible with gamma distribution model (GTR+G+I) [24] with a bootstrap support of 1000 replications using the MEGA X program [25]. A minimum spanning haplotype network(s) of AkInt3 was generated using Network v.5.0.11 (http://www.fluxus-engineering.com/ accessed on 12 August 2024) based on the median-joining algorithm [26].

## 3. Results

**Figure 1:**
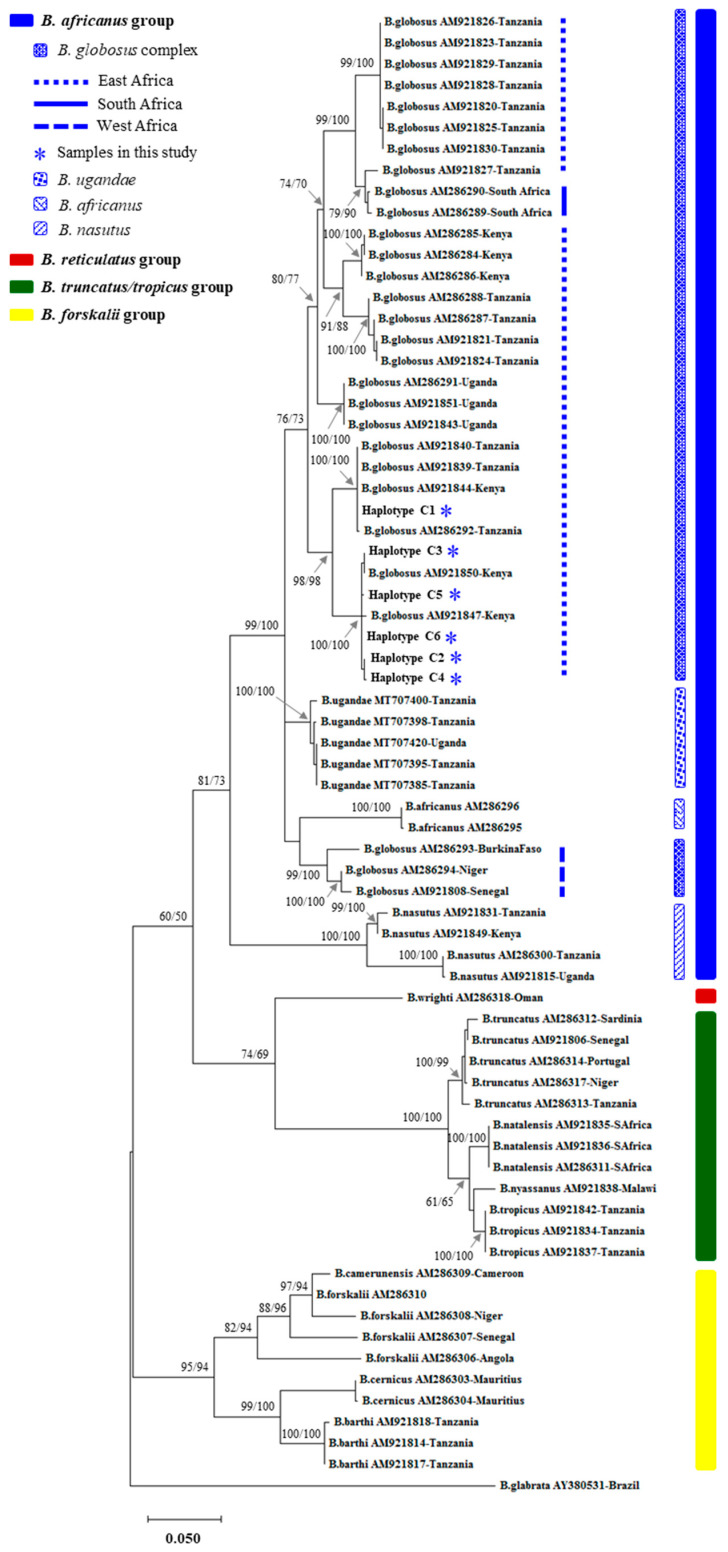
Maximum likelihood tree of the *Bulinus* snails constructed based on *COI* sequences. Nodal supports are the bootstrap values generated by neighbor joining followed by those from maximum likelihood analyses. The star represents six *COI* haplotypes (C1–C6) of *B. globosus* examined in this study (see more details in Table 1).

Based on cercarial shedding, we identified one *B. globosus* snail (1.2%) infected with *S. haematobium*. This identification was confirmed by *COI* genotyping with 99% similarity to *COI* sequences of *S. haematobium* deposited in GenBank. The *COI* sequence of *S. haematobium* examined in our study has been deposited in GenBank under the accession number PQ764858. The 81 *COI* sequences of *B. globosus* examined in this study showed the highest similarity with *B. globosus* samples deposited in the GenBank database, which were also closely aligned with some sequences of *B. globosus* from Tanzania and Kenya (East Africa) of the *B. globosus* complex (Figure 1). There were 21 nucleotide variable sites (1 singleton variable and 20 parsimony informative sites) from the 632 bp of the *COI* sequence that we examined. These variations were used to classify the 81 *B. globosus* sequences into six *COI* haplotypes, namely C1–C6 (Table 1). Haplotype C1 was commonly found in all sampling localities, whereas haplotypes C2–C4 and C5–C6 were specifically detected in the Maelinane (ML) and Mwachinga (MC) localities, respectively (Table 1). Haplotype diversity and nucleotide diversity based on *COI* ranged between 0.000 ± 0.000 and 0.703 ± 0.046 and between 0.000 ± 0.000 and 0.0154 ± 0.0019, respectively (Table 2).

From the AkInt3 sequence analyses, we found that 39 and 14 snails were homozygous and heterozygous, respectively (Table 1). After DNA cloning and sequencing of heterozygous snails, a total of 128 sequences of AkInt3 were obtained. Comparisons of all sequences found 86 variable sites, which were classified as 45 singleton variable sites and 41 parsimony informative sites (Table S1). Based on these variations, 44 AkInt3 haplotypes (H1–H44) were generated. Three common (shared) haplotypes, i.e., H1, H14, and H31 were detected in all three localities, whereas the other haplotypes were uniquely detected in one specific geographical area. Haplotype diversity and nucleotide diversity based on AkInt3 ranged between 0.838 ± 0.036 and 0.855 ± 0.033 and between 0.0203 ± 0.0019 and 0.0221 ± 0.0021, respectively (Table 2).

**Figure 2:**
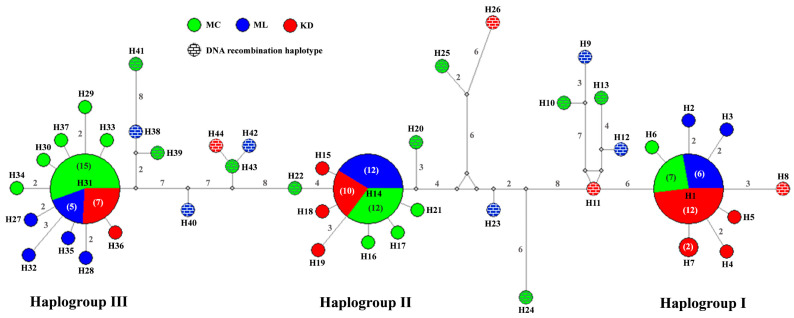
Minimum spanning haplotype network generated from the sequences of 44 AkInt3-classified haplotypes (H1–H44) of *Bulinus globosus* into three haplogroups, i.e., haplogroup I, II, and III. The color in each circle represents the locality. Numbers in each branch represent the mutational step number, in which branch with no number indicates one mutational step. The numbers in brackets in each circle represent the number of sequences contained in a particular circle, while no number indicates that only one sequence was contained. Small white dots are median vectors.

The AkInt3 haplotype network separated *B. globosus* into three main haplogroups, i.e., haplogroup I, II, and III. The AkInt3 sequence was subsequently defined into three fragments that corresponded to specific detection in each haplogroup. Thus, each haplotype consisted of either DNA fragment(s) found only in a particular haplogroup or combined DNA fragments between haplogroups (DNA recombination haplotype) (Table S1). For instance, haplotypes H1–H7, H14–H19, and H27–H37 contained DNA fragments only found in haplogroups I, II, and III, respectively. However, DNA recombination haplotypes were found in several haplotypes that aligned between the three main haplogroups of the haplotype network (Figure 2). For example, H8, H11, H12, H13, H23, and H24 were DNA recombinant haplotypes between haplogroup I and II, whereas H9, H10, H38, H39, and H41 were DNA recombinant haplotypes between haplogroup I and III, while H20, H22, H25, H26, H40, H42, H43, and H44 were DNA recombinant haplotypes between haplogroup II and III (Figure 2).

## 4. Discussion

Our study confirms that all of the snails analyzed were molecularly identified as *B. globosus* using *COI* genotyping. They were all clustered in the East Africa group of the *B. globosus* complex [3]. This classification was supported by the *COI* genotyping. Even though our investigation was conducted at a micro-scale, we found extremely high nucleotide variation in the AkInt3 region when compared within and between three different populations in Kenya. On the other hand, the *COI* sequence showed a nucleotide variation within *B. globosus* populations in Kenya that was lower than the variation in the AkInt3 region. Hence, the AkInt3 region can provide a more comprehensive assessment of the levels of intra-specific genetic variation within and between the populations of the *B. globosus* complex from Kenya, highlighting its utility in capturing finer-scale genetic diversity.

In addition, the observed heterozygosity in some snails revealed DNA recombination between different AkInt3 haplogroups, providing evidence that the majority of *B. globosus* snails in Kenya reproduce by cross-fertilization, which further supports previous observations [7,9]. The results of our study uncovered three haplotypes in a few heterozygous specimens. A previous study has detected similar cross-fertilization in *Fasciola* spp. [12]. Polyploidy has previously been observed in several species of the *B. truncates/tropicus* complex [27]. Thus, based on our results, the preferential mode of reproduction was cross-fertilization, which reduces self-fertilization depression in fitness [7,9], and it is possible that the *B. globosus* populations in Kenya contained snails showing polyploidy. To the best of our knowledge, there is only one previous report of karyotype analysis of *B. globosus* compared with other related species since 1971, where only diploidy (*n* = 18) was observed [28]. Comprehensive karyotype analyses of populations of the *B. globosus* complex in Africa need to be conducted to confirm discrepancies in ploidy levels.

We found high nucleotide polymorphism in an intron (AkInt3) region of *B. globosus* which, together with the detection of heterozygous samples, revealed DNA recombination haplotypes (i.e., the combination between DNA fragments of different genetically distinct haplogroups). Our results underscored that the AkInt3 region has promise as an alternative genetic marker for further population genetic investigations of the *B. globosus* complex. Furthermore, AkInt3 primers that were designed in our study have the potential to be used for the cross-amplification of other related species in the *B. africanus* group. This approach has previously successfully used the same primer set to amplify intron regions of different species of trematodes [12,13], as well as *Bithynia* snails [15]. Hence, AkInt3 may prove useful for the genetic investigations of other species within the *B. africanus* group. The haplotype network of AkInt3 delineates three genetically distinct haplogroups within *B. globosus* populations in Kenya. The observed genetic differences, with mutational steps of generally less than 10, have been used to indicate intra-specific genetic variation in other freshwater snails, such as *Bithynia* [29] and *Oncomelania* [30]. Our findings suggest that the *B. globosus* populations in Kenya represent a “species complex” containing high levels of genetic variation, which were classified into at least three very different genetically distinct groups.

The high genetic variability observed in *Bulinus* snails in Kenya has important implications for understanding genetic variation in *S. haematobium*, the trematode parasite of which these snails act as intermediate hosts of. In areas where both the parasite and snail host show high levels of genetic diversity, coevolutionary pressures are likely at play, potentially driving local adaptations in the parasite to match specific host genotypes. Studies suggest that *S. haematobium* populations may exhibit genetic variation that corresponds to the genetic structure of their *Bulinus* hosts, as parasite populations adapt to infect specific host lineages more effectively [31,32]. This host–parasite genetic interplay can lead to local adaptation, where parasite infectivity is optimized for the prevalent host genotypes in a given area. Such genetic diversity in *Bulinus* could result in varied infection rates and possibly influence the transmission dynamics of schistosomiasis across regions in Kenya. In this study, we found one snail from Mwachinga (MC_2-1) infected with *S. haematobium*. Interestingly, infection of snail sample MC_2-1 was a common haplotype in both *COI* and AkInt3, namely C1 and H14, respectively. This finding could have important implications for understanding the genetic factors that influence the transmission dynamics of schistosomiasis in this region and may contribute to identifying genetic markers associated with increased susceptibility to infection in *B. globosus*. Further studies are needed to explore the potential of these haplotypes as genetic markers for schistosome susceptibility in *Bulinus* snails.

Additionally, high host variability may serve as a genetic buffer, potentially complicating parasite establishment and persistence, which could provide a basis for schistosomiasis control efforts that target genetic diversity in host populations [33]. This relationship between host and parasite genetic diversity underscores the importance of considering both host and parasite genetics in managing schistosomiasis [34]. To fully understand the evolutionary and systematic relationships of the *B. globosus* complex, as well as the co-adaptation between this snail and its parasites, comprehensive investigations of the genetic differences, genetic structure, population genetics; the morphology, biology, and ecology of the *B. globosus* populations; and its parasites, especially *S. haematobium* in Kenya and throughout the African continent, are further required.

## 5. Conclusions

Our study reveals substantial genetic diversity within *B. globosus* populations in Kenya, demonstrating that the AkInt3 intron region is a powerful molecular marker for examining intra-specific variation. The AkInt3 region exhibited greater nucleotide polymorphism than *COI* sequences, providing a more distinct view of the genetic structure. Evidence of DNA recombination between AkInt3 haplogroups suggests cross-fertilization as a key reproductive strategy, potentially mitigating inbreeding depression, with possible polyploidy indicating further genetic complexity. Additionally, the detection of distinct haplogroups within Kenyan populations suggests a complex species assemblage, underscoring the need for more genetic and ecological research across Africa to better understand *B. globosus* evolution, its role in disease transmission, and implications for schistosomiasis control.

## Figures and Tables

**Table 1:** Details of the geographical sample localities, *COI* and AkInt3 haplotype analyses, heterozygosity, and DNA fragments recombination.

Locality (Sample Number)	Sample Code	*COI*		AkInt3			
		Haplotypes		Homo	Het	Haplotypes	DNA Fragments *
Kinango (26)	KD_1-1	C1		✓		H14	II
Habitat: Kinango dam	KD_1-2	C1		✓		H14	II
Lat, long: −4.13636, 39.31000	KD_1-3	C1			✓	H5	I
Collection date: 20 April 2011						H14	II
	KD_1-4	C1			✓	H1	I
						H14	II
	KD_5-1	C1			✓	H1	I
						H11	I + II
						H14	II
	KD_5-2	C1			✓	H8	I + II
						H14	II
	KD_5-3	C1			✓	H14	II
						H44	III + II
	KD_5-4	C1			✓	H4	I
						H14	II
	KD_5-5	C1		✓		H1	I
	KD_5-6	C1		✓		H1	I
	KD_5-7	C1		✓		H31	III
	KD_5-8	C1			✓	H26	II+III
						H31	III
	KD_5-9	C1		✓		H1	I
	KD_5-10	C1			✓	H14	II
						H31	III
	KD_5-11	C1		✓		H1	I
	KD_5A	C1		✓		H1	I
	KD_5B	C1			✓	H1	I
						H31	III
	KD_5C	C1			✓	H15	II
						H36	III
	KD_5D	C1			✓	H1	I
						H31	III
	KD_7-1	C1			✓	H1	I
						H18	II
	KD_7-2	C1		✓		H1	I
	KD_7-3	C1			✓	H7	I
						H19	II
	KD_7-4	C1		✓		H31	III
	KD_7-6	C1			✓	H7	I
						H14	II
	KD_7-8	C1		✓		H31	III
	KD_7-9	C1		✓		H14	II
Mwachinga (31)	MC_2-1	C1		✓		H14	II
Habitat: Natural reservoir	MC_4-1	C1		✓		H14	II
Lat, long: −4.1166, 39.3833	MC_4-2	C1			✓	H1	I
Collection date: 18 April 2011						H31	III
	MC_4-3	C1			✓	H14	II
						H31	III
	MC_4-4	C5		✓		H31	III
	MC_4-5	C1		✓		H14	II
	MC_4-6	C1			✓	H31	III
						H43	III + II
	MC_4-7	C1			✓	H14	II
						H31	III
	MC_4-8	C6			✓	H1	I
						H39	III + I
	MC_4-9	C1			✓	H1	I
						H31	III
	MC_4-10	C1			✓	H21	II
						H24	II+I
	MC_4-11	C1			✓	H16	II
						H33	III
						H34	III
	MC_4-12	C1			✓	H14	II
						H30	III
	MC_4-13	C1			✓	H6	I
						H31	III
	MC_4-14	C1		✓		H14	II
	MC_4-15	C1			✓	H22	II + III
						H31	III
	MC_4-16	C1			✓	H14	II
						H20	II + III
						H25	II + III
	MC_4-17	C1		✓		H14	II
	MC_4-18	C6		✓		H14	II
	MC_6-1	C1		✓		H1	I
	MC_6-2	C1		✓		H31	III
	MC_6-3	C1		✓		H31	III
	MC_6-4	C1		✓		H31	III
	MC_6-5	C6			✓	H14	II
						H29	III
	MC_6-6	C1			✓	H1	I
						H31	III
	MC_6-7	C6			✓	H1	I
						H31	III
						H41	III + I
	MC_6-8	C6			✓	H1	I
						H13	I + II
						H17	II
	MC_6-13	C1			✓	H10	I + III
						H37	III
	MC_6-15	C1		✓		H31	III
	MC_6-16	C1		✓		H14	II
	MC_6-18	C1		✓		H31	III
Maelinane (24)	ML_3-1	C2			✓	H1	I
Habitat: Natural reservoir						H14	II
Lat, long: −4.0961, 39.4243	ML_3-2	C3		✓		H31	III
Collection date: 18 April 2011	ML_3-3	C3			✓	H14	II
						H40	III + II
	ML_3-4	C3		✓		H14	II
	ML_3-5	C2		✓		H14	II
	ML_3-6	C2		✓		H1	I
	ML_3-7	C4			✓	H1	I
						H31	III
	ML_3-8	C2		✓		H42	III + II
	ML_3-9	C1		✓		H1	I
	ML_3-10	C2		✓		H1	I
	ML_3-11	C3			✓	H9	I + III
						H27	III
	ML_3-12	C2		✓		H31	III
	ML_3-13	C1			✓	H14	II
						H23	II + I
	ML_3-14	C2			✓	H12	I + II
						H14	II
	ML_3-15	C1			✓	H3	I
						H28	III
	ML_3-16	C2		✓		H14	II
	ML_3-17	C3			✓	H2	I
						H14	II
	ML_3-18	C2			✓	H1	I
						H31	III
	ML_3-19	C1			✓	H32	III
						H38	III + I
	ML_3-20	C1			✓	H31	III
						H35	III
	ML_L1	C1		✓		H14	II
	ML_L2	C1		✓		H14	II
	ML_L3	C1		✓		H14	II
	ML_L4	C1		✓		H14	II

* DNA fragments found in: I, haplogroup I; II, haplogroup II; III, haplogroup III (see more details in Table S1). Sample codes indicated in bold contained three AkInt3 haplotypes within a particular heterozygous sample. Homo, homozygous; Het, heterozygous.

**Table 2:** Diversity indices of *COI* and AkInt3 sequences of three *Bulinus globosus* populations in Kenya.

Populations (Code)	*n*	*COI*						AkInt3				
		S	H	Uh	Hd *±* SD	π ± SD		S	H	Uh	Hd *±* SD	π ± SD
Kinango dam (KD)	26	0	1	0	0.000 ± 0.000	0.0000 ± 0.0000		52	14	12	0.838 ± 0.036	0.0203 ± 0.0019
Mwachinga (MC)	31	18	3	2	0.288 ± 0.097	0.0079 ± 0.0017		60	21	18	0.855 ± 0.033	0.0213 ± 0.0020
Maelinane (ML)	24	21	4	3	0.703 ± 0.046	0.0154 ± 0.0019		56	15	11	0.847 ± 0.046	0.0221 ± 0.0021
Total	81	21	6	5	0.419 ± 0.065	0.0113 ± 0.0017		86	44	41	0.851 ± 0.019	0.0212 ± 0.0002

*n*, sample size; S, segregation site; H, number of haplotypes; Uh, unique haplotype; Hd, haplotype diversity; π, nucleotide diversity; SD, standard deviation.

## Data Availability

All data are available upon request.

## Supplementary Materials

The following supporting information can be downloaded at: https://www.mdpi.com/article/10.3390/biology14010053/s1, Table S1: Variable nucleotide positions comparison between 44 AkInt3 haplotypes of *Bulinus globosus* in Kenya.
